# Development and evaluation of a simulation-based transition to clerkship course

**DOI:** 10.1007/s40037-020-00590-4

**Published:** 2020-05-26

**Authors:** Jared P. Austin, Mark Baskerville, Tracy Bumsted, Leslie Haedinger, Stephanie Nonas, Eugen Pohoata, Meghan Rogers, Megan Spickerman, Philippe Thuillier, Suzanne H. Mitchell

**Affiliations:** 1 Department of Pediatrics, Oregon Health & Science University, Portland, OR USA; 2 Department of Anesthesiology and Perioperative Medicine, Oregon Health & Science University, Portland, OR USA; 3 School of Medicine, Oregon Health & Science University, Portland, OR USA; 4 Department of Medicine, Oregon Health & Science University, Portland, OR USA; 5 Department of Dermatology, Oregon Health & Science University, Portland, OR USA; 6 Departments of Behavioral Neuroscience, Psychiatry and the Oregon Institute of Occupational Health Sciences, Oregon Health & Science University, Portland, OR USA

**Keywords:** Clerkship, Transition, Simulation, Objective structured clinical examination

## Abstract

**Background:**

Transition to clerkship courses bridge the curricular gap between preclinical and clinical medical education. However, despite the use of simulation-based teaching techniques in other aspects of medical training, these techniques have not been adequately described in transition courses. We describe the development, structure and evaluation of a simulation-based transition to clerkship course.

**Approach:**

Beginning in 2012, our institution embarked upon an extensive curricular transformation geared toward competency-based education. As part of this effort, a group of 12 educators designed, developed and implemented a simulation-based transition course. The course curriculum involved seven goals, centered around the 13 Association of American Medical Colleges Core Entrustable Professional Activities for entering residency. Instructional techniques included high-fidelity simulation, and small and large group didactics. Student competency was determined through a simulation-based inpatient-outpatient objective structured clinical examination, with real-time feedback and remediation. The effectiveness of the course was assessed through a mixed methods approach involving pre- and post-course surveys and a focus group.

**Evaluation:**

Of 166 students, 152 (91.6%) completed both pre- and post-course surveys, and nine students participated in the focus group. Students reported significant improvements in 21 out of 22 course objectives. Qualitative analysis revealed three key themes: learning environment, faculty engagement and collegiality. The main challenge to executing the course was procuring adequate faculty, material and facility resources.

**Reflection:**

This simulation-based, resource-heavy transition course achieved its educational objectives and provided a safe, supportive learning environment for practicing and refining clinical skills.

**Electronic supplementary material:**

The online version of this article (10.1007/s40037-020-00590-4) contains supplementary material, which is available to authorized users.

## Background

After calls for reform in the early 20th century, the Flexner Report standardized undergraduate medical education in the United States [1]. Based on Flexner’s 2 × 2 “Hopkins” model, the first two ‘preclinical’ years are spent primarily in the classroom, while the second two ‘clinical’ years are focused on clinic- and hospital-based rotations, or clerkships. This front-loaded didactic model came at the expense of meaningful interaction with patients prior to clerkships. Consequently, the lack of clinical experience early in training has made the transition period to the clinical environment stressful for learners and clerkship directors [2, 3]. To alleviate this stress and to better prepare students for the clinical realm, many medical schools have implemented a transition to clerkship course [4–6].

Traditional transition to clerkship courses focus largely on workplace-based skills, such as oral presentations, hospital and clinic logistics, student roles and responsibilities, and more recently, use of the electronic medical record [4, 5]. In a large 2010 study of US and Canadian medical schools, 88% of schools reported having a transition course [4]. However, only 35% of these courses issued a course grade (including pass/fail), and only 41% included evaluations of student performance. Didactic sessions were present in 98% of courses, hands-on practice in 74%, and clinical immersion in only 21%. While this study did not define the type of ‘hands-on’ training, simulation-based teaching techniques were likely used in many of the courses. The authors concluded that transition to clerkship courses should incorporate real clinical settings, emphasize hands-on practice of clinical skills, and formalize evaluations.

Transition to residency and graduate medical education courses have leveraged high- and low-fidelity simulation extensively over the past 20 years [7–9]. However, comparable literature is lacking for the use of simulation in transition courses. Since medical students are often excluded from many aspects of primary patient management (e.g. procedures), simulation provides a modality to develop important clinical skills without risk to patients. Additionally, given patient safety concerns—particularly involving vulnerable populations—some have gone as far as mandating simulation-based education as an ethical imperative [10]. Regardless, multiple studies involving many different types of learners have shown compelling benefits from simulation-based education and, hence, incorporation of simulation-based education into transition courses seems justified [9, 11]. Additionally, simulation provides the opportunity for deliberate practice, which has been described as an essential element for the development of expertise [12]. This concept was displayed in a recent article by Ryan et al., where student performance on clinical rotations significantly improved following implementation of a transition course involving a significant simulation component [13].

Accordingly, during a comprehensive curriculum transformation of undergraduate medical education at our institution, we incorporated high-fidelity simulation into a new transition to clerkship course. This new course serves as a ‘gateway’ between preclinical and clinical phases of training, preparing students for their expanded roles on clinical rotations. The purpose of this report is to describe the development, structure and evaluation of this course.

## Approach

Beginning in 2012, our institution embarked upon an extensive transformation of the undergraduate medical curriculum [14]. The curriculum was divided into two phases: foundational (first 18 months) and clinical experiences (19–48 months). Although weekly clinical skills labs and preceptorships were incorporated during the foundational stage, curriculum developers perceived a need for a transition course to bridge the curricular gap between the foundational and clinical phases of training, as well as to provide learners with a psychological demarcation of their transition to full-time, immersive clinical training. Curriculum developers used the Association of American Medical Colleges (AAMC) Core Entrustable Professional Activities (EPAs)[15, 16] framework, as well as evidence from transition courses, simulation-based education [4, 5, 9], and the expertise literature [12], in designing the course.

The first transition to clerkship course was held in 2016, and has evolved since then with iterative improvements adopted from learner and teacher feedback. Specifically, we have incorporated more senior medical student teachers into teaching sessions and reduced the number of times the course is offered from twice to once a year, thus saving considerable resources. Two weeks in duration, the course has seven goals, each with associated objectives and educational and assessment strategies (Tab. 1). Instruction for each goal involves a combination of pre-work (e.g. videos, readings, reflections), and large and small group sessions, including high-fidelity simulation. Students are taught the content during the first week, and are assessed during the second week with a comprehensive inpatient-outpatient objective structured clinical examination (OSCE) (Tab. 1 and Supplemental Tab. 1). The course is graded as pass/fail.

**Table 1:** Description of transition to clerkship course

Goal	EPA^a^ links	Objectives	Learning sessions	Assessment
Goal 1: Recognize a patient requiring urgent or emergent care and demonstrate the ability to appropriately call for help and initiate basic life-saving maneuvers	– EPA 10	– Demonstrate the ability to perform effective cardiopulmonary resuscitation, including basic airway management, and proper technique in performing jaw thrust, chin tilt, and bag-mask ventilation – Demonstrate how and when to activate rapid response, code blue, or emergency services for behavioral and medical crises, and describe which members of the interprofessional team are included in each of these groups – Recognize normal and unstable vital signs and variations that might be expected, based on patient and disease-specific factors – Recognize physical exam and laboratory findings indicative of clinical instability	– Large group, interactive session on identifying and responding to unstable vital signs – Small group, high-fidelity simulation: students rotate through 3 stations—adult patient with cardiac arrest; pediatric patient with fever, sepsis, and seizure; and an unstable psychiatric patient	– Inpatient/outpatient Observed Structured Clinical Examination (OSCE), using standardized rubrics^b^
Goal 2: Communicate effectively and efficiently with patients, colleagues, and staff in both oral and written form	– EPAs 5, 6, 8	– Demonstrate communication skills necessary to competent clinical care, including giving oral presentations, transition of care handoffs and describing how and when to call for consults – Use the electronic medical record to gather information on an assigned patient, and prepare and give an oral presentation – Demonstrate giving and receiving handoffs using closed-loop communication in various simulated transition settings	– Small group, high-fidelity activity: students are assigned a patient in the “practice” electronic medical record. They must access information for the patient and prepare and give an oral presentation and handoff on the patient. Facilitators and classmates provide feedback	– Inpatient/outpatient OSCE
Goal 3: Demonstrate standard patient and personal protective equipment (PPE) safety measures appropriate to the healthcare environment	– EPA 12	– Demonstrate proper scrubbing, gowning, gloving, sterile technique, and management of sharps and contaminated materials	– Small group, high-fidelity simulation: students rotate through 2 stations: infection control/PPE and scrubbing, gowning, gloving in a simulated operating theater	– Inpatient/outpatient OSCE
Goal 4: Recognize gaps in knowledge, skills and attitudes, and identify and begin to utilize effective strategies for lifelong learning	– EPA 7	– Recognize one’s own limitations and strengths – Describe several strategies to learn on your own and share knowledge with others, including searching primary literature to answer a clinical or science question about disease pathology, and generating questions to extend your knowledge of the foundational science related to clinical problems – Use information technology to find and apply knowledge-based information to healthcare for patients and populations – Explain components of formative assessments and clinical expectations during the clinical experiences	– Large group didactic session on generating and answering clinical questions. Overview of small group presentation expectations – Large group didactic session with a panel of clerkship directors – Small group activity: students deliver an evidence-based short presentation on a chosen clinical topic to faculty grader and staff	– Inpatient/outpatient OSCE – Small group oral presentations
Goal 5: Understand the core professional, ethical, legal, and clinical expectations of students, regardless of healthcare environment or discipline	– EPAs 11, 13	– Demonstrate a grasp of ethical and medicolegal topics, including informed consent, malpractice, and confidentiality	– Large group, high-fidelity simulated morbidity and mortality conference. Session involves analyzing a plane crash as well as case of medical neglect, with discussion on legal and ethical components, and root-cause analyses	– Inpatient/outpatient OSCE
Goal 6: Understand and employ effective strategies to balance personal wellness alongside clinical duties	– None	– Discuss achieving and maintaining student wellness during rotations, and how and where you will seek help when you feel overwhelmed – Plan a strategy to work respectfully and effectively in the clinical environment	– Large group, interactive session run by university wellness leaders, focused on strategies to maintain wellness on rotations – Large group, interactive session run by an associate dean on student mistreatment – Individual activity: Reflection on personal wellness and strategies to maintain wellness on clinical rotations	– Not directly assessed
Goal 7: Participate in an in-situ shadowing experience, and submit and share reflections in a small group	– EPA 9	– Observe the dynamics of a clinical team during rounds – Plan strategies to work respectfully and effectively in the clinical environment	– Individual activity: students participate in a clinical ward shadowing experience to observe various roles on the interprofessional work team – Small group activity: students participate in a post shadowing debrief session, led by students and faculty	– Small group session with faculty and senior medical students

*EPA* entrustable professional activity, *PPE* personal protective equipment

^a^Association of American Medical Colleges Core Entrustable Professional Activities (EPAs)

^b^See standardized grading rubrics: Supplemental Figs. 2, 3 and 4

The OSCE involves both adult and pediatric cases that progress between inpatient and outpatient settings (Supplemental Tab. 1 and Supplemental Fig. 1). Whereas in the first week of the course skills associated with each goal are taught individually, in the OSCE these skills are integrated into a continuous case. Students begin the OSCE in either the outpatient or inpatient setting, and then proceed to the other setting upon scenario completion, following the same simulated patient (Supplemental Fig. 1). Standardized rubrics are used to assess performance in both the inpatient (Supplemental Fig. 2) and outpatient (Supplemental Figs. 3 and 4) scenarios. Students are graded by faculty and standardized patients. Students who do not achieve a passing score for any of the components of the OSCE using the standardized rubrics are remediated in real-time. Remediation is done by a faculty member or the course director. Students must display adequate knowledge, skills or attitudes in the particular element(s) they failed. At the end of the inpatient portion of the OSCE, students are debriefed in small groups by an instructor and key learning points are emphasized.

In 2019, students taking the course completed a pre- and post-course survey to assess comfort level with the objectives of the course. The post-course survey also solicited open feedback about the course in a free-text format. Because data were ordinal and many measures exhibited pre- and post-changes in skew, pre- and post-course quantitative results were calculated using Wilcoxon signed-rank tests using SPSS (IBM, version 25). Qualitative analysis was performed by two senior authors using directed qualitative content analysis [17]. Themes were developed independently, then combined and collated. Exemplar comments were chosen to emphasize each theme. Nine months after the completion of the course, a 1-hour focus group was conducted by two senior authors to validate themes from the qualitative analysis.

Our Institutional Review Board deemed the study exempt from review.

## Evaluation

Of 166 medical students enrolled in transition to clerkship in 2019, 152 completed both pre- and post-course surveys for a response rate of 91.6%. Nine students (5 males, 4 females) participated in the post-course focus group, and two sets of field notes were taken independently by two senior authors, collated and used for validation and clarification of qualitative themes. Post-course means were significantly higher than pre-course means for 21 out of 22 objectives, reflecting improved confidence in skills taught during the course (Supplemental Tab. 2).

Qualitative content analysis revealed three main themes: learning environment, faculty engagement and collegiality (Tab. 2). Within the learning environment theme, 4 sub-themes were identified: hands-on/active learning, pertinent skills for clinical practice, pertinent skills for the clinical environment, and course/session structure.

**Table 2:** Qualitative content analysis of transition to clerkship: major themes, sub-themes and exemplar comments

Major themes	Sub-themes	Exemplar comments
*Learning environment*	Hands-on, active learning	– “I really found the active learning valuable. The whole range of active learning (from presenting/listening to peer presentations all the way to working with Sim Center models) were helpful”
	Pertinent skills for clinical practice	– “I really appreciated the training on unstable patients and the use of the simulation lab. Although I felt uncomfortable not knowing what was “next”, the unpredictability of the scenarios allowed me to better prepare for my time in the hospital and clinics” – “I particularly enjoyed the clinical sessions where we got to practice in the simulation lab. I really thought these were helpful in the pediatric case, because I really had no idea what I should be looking for in terms of emergencies with infants” – “It was super helpful to have practice with real-world situations. I appreciated the chance to learn and practice in a simulation”
	Pertinent skills for the clinical environment	– “The strengths were the simulations and practical skills: scrubbing, PPE, oral presentations, etc” – “Scrubbing in, donning and doffing PPE, practicing handoffs and presentations were all extremely helpful!”
	Course/session structure	– “I think having a week of practice scenarios followed by a week of testing was really helpful” – “I loved the way this course was structured and the different skills we were taught. I especially liked the unstable patient/CPR portion … the main thing that helped the most was the instructors having us do the scenarios over and over” – “I absolutely loved this course. It was how I envisioned much of medical school would be. Being thrust into an uncomfortable situation and asked to repeat a task over and over gave us a great opportunity to critique ourselves, improve, and become comfortable in a chaotic environment”
*Faculty engagement*		– “Really great teachers and passionate about what they were teaching” – “Everyone was respectful and gave really constructive feedback during the sessions that I think/hope will be valuable for clinicals!”
*Collegiality*		– “I also really appreciated working in small groups where I could get to know my new classmates better and the tasks (i.e., presentations, oral presentation/handoff practice) seemed a lot lower- risk” – “I liked the group sizes. I felt like I got to engage with my peers better”

*PPE* personal protective equipment, *CPR* cardiopulmonary resuscitation

## Reflection

Curricular reform, directed toward competency-based educational outcomes, is a current priority of many medical schools, including ours [14]. The utilization of simulation-based teaching techniques in this course, including the use of a multi-structured OSCE, was helpful in teaching and assessing competency in key areas of clinical practice, and providing real-time feedback and remediation. The course was well received by learners, who demonstrated increased confidence levels in most objectives, and remarked positively on the use of simulation techniques in teaching content. Students also reported positively on the supportive role of faculty and the ability to connect with peers through small group sessions.

The use of a high-stakes, multi-structured OSCE was unique to our course, and helped address the concern raised by O’Brien et al. in terms of the lack of student evaluations in 41% of transition courses [4]. In line with the aims of the AAMC Core EPA project [16], the OSCE ensured that students could display a certain threshold of competence in key aspects of clinical care prior to entering the clinical environment, thus aiding in a successful transition. Students who did not reach passing thresholds on the OSCE were provided immediate feedback and remediation in a nonjudgmental environment with the opportunity to repeat testing until competence had been demonstrated. As confirmed by feedback during the focus group, the pass/fail grading structure, with real time remediation, helped decrease anxiety among students, and enhanced learning in a ‘safe’ environment. Furthermore, the use of evidence-based evaluation tools was helpful in standardizing formative and summative evaluations of student performance, particularly in regards to oral presentations [18].

Given the nature of high-fidelity simulation, our course requires significant faculty, material and facility resources. For example, the 2019 course utilized 66 different facilitators, 5 high-fidelity simulation rooms for 6–8 h per day over 8 days, and 12 standardized patients, each with a simulation examination room, for 8 h per day over 4 days. Since this course was designed within the construct of our school’s curriculum transformation, we have been fortunate to receive considerable support from our educational administration in meeting these needs. However, recruitment of faculty for the many teaching components remains a challenge. To address this challenge, we have begun using senior medical students to teach some elements, including scrubbing/gowning/gloving, and giving oral presentations. In the future, we plan to implement a specific ‘teaching elective’ for senior medical students, which will allow them a more formal role in teaching and modifying the course to suit the needs of learners. In institutions with resource limitations, many of the sessions could be conducted with lower fidelity technology and/or incorporate more peer-to-peer teaching and team-based learning approaches.

Our study had several important limitations. First, since the study was conducted within a single medical school, the results may not be generalizable to other settings. Second, quantitative outcomes were based on self-assessment of student comfort level with individual tasks and, therefore, may be less reliable than objective measures of competence. Third, outcomes were recorded for one academic year and, therefore, sampling bias may affect results. Sampling over multiple years would also help determine the efficacy of iterative changes made to the course. Finally, beyond the focus group, follow-up data were not obtained to examine whether the course facilitated students’ performance in their clinical rotations. Future research into clinical performance outcomes from transition to clerkship courses is needed.

## Caption Electronic Supplementary Material

Supplemental Fig. 1: Student flow chart for Objective Structured Clinical Examination (OSCE)

Supplemental Fig. 2: Inpatient OSCE Checklist

Supplemental Fig. 3: Oral Case Presentation Rating Scale for Outpatient OSCE

Supplemental Fig. 4: Outpatient OSCE Encounter Checklist by Standardized Patient

Supplemental Table 1: Description of Transition to Clerkship Objective Structured Clinical Examination (OSCE)

Supplemental Table 2: Means (and Standard Deviations) for Student Scores on the Self-Assessment Survey of Transition to Clerkship Objectives: Pre-versus Post-Course
