# Supplementary material for: Development and evaluation of a simulation-based transition to clerkship course
Source: Perspect Med Educ. 2020 May 26;9(6):379–84. doi: 10.1007/s40037-020-00590-4 (PMC7718359; doi:10.1007/s40037-020-00590-4)
Supplement: Supplementary file 1 — Supplemental Fig. 1: Student flow chart for Objective Structured Clinical Examination (OSCE) [file 40037_2020_590_MOESM1_ESM.docx]

**Inpatient start**

Adult Inpatient case **OR** Pediatric Inpatient case

(15 minutes)

Adult Post-Outpatient case **AND** Pediatric Post-Outpatient case

(12 min encounter, 8 min preparation, 20 minute presentation)

Supplemental Figure 1: Student flow chart for Objective Structured Clinical Examination (OSCE)

**Outpatient start**

Adult Pre-Outpatient case **AND** Pediatric Pre-Outpatient case

(12 min encounter, 8 min preparation, 20 minute presentation)

Adult Inpatient case **OR** Pediatric Inpatient case

(15 minutes)
