# Supplementary material for: Development and evaluation of a simulation-based transition to clerkship course
Source: Perspect Med Educ. 2020 May 26;9(6):379–84. doi: 10.1007/s40037-020-00590-4 (PMC7718359; doi:10.1007/s40037-020-00590-4)
Supplement: Supplementary file 2 — Supplemental Fig. 2: Inpatient OSCE Checklist [file 40037_2020_590_MOESM2_ESM.docx]

| **OSCE- Inpatient** | **No  (0)** | **Yes**  **(1)** | | **Comments** |
| --- | --- | --- | --- | --- |
| 1. Recognize an unstable patient |  | |  |  |
| 1. Appropriately call for help |  | |  |  |
| 1. Demonstrate basic airway management |  | |  |  |
| 1. Appropriately don and doff PPE without contamination, or demonstrate proper scrubbing, gowning, gloving technique |  | |  |  |
| 1. Demonstrate proper sharps safety |  | |  |  |
| 1. Research and find answers to a clinical question |  | |  |  |
| 1. Recognize limits of practice |  | |  |  |
| 1. Maintain confidentiality |  | |  |  |
| 1. Articulate the components of informed consent (PARQ) |  | |  |  |
| 1. Give or receive a handoff |  | |  |  |

Additional comments:

Total points ________/10 Passing = 7/10

Supplemental Figure 2: Inpatient OSCE Checklist
