# Supplementary material for: Development and evaluation of a simulation-based transition to clerkship course
Source: Perspect Med Educ. 2020 May 26;9(6):379–84. doi: 10.1007/s40037-020-00590-4 (PMC7718359; doi:10.1007/s40037-020-00590-4)
Supplement: Supplementary file 3 — Supplemental Fig. 3: Oral Case Presentation Rating Scale for Outpatient OSCE [file 40037_2020_590_MOESM3_ESM.docx]

Student ______________________________ Evaluator______________________ Date_________________

| **SUBJECTIVE and OBJECTIVE** | **Needs Improvement**  **(0)** | **Adequate**  **(1)** | **Excellent**  **(2)** | **Comments *(optional)*** |
| --- | --- | --- | --- | --- |
| 1. *Chief concern noted either before HPI or as part of introductory sentence* | No chief concern noted  □ | Chief concern mentioned  □ | Chief concern clear  □ |  |
| 1. *HPI starts with clear patient introduction including patient’s age, sex, pertinent active medical problems and reason for seeking care* | No introductory sentence  □ | Intro included most of the pertinent information  □ | Intro painted a clear picture of patient  □ |  |
| 1. *HPI is organized so that chronology of important events and symptoms is clear* | Sequence of events is unclear □ | Sequence of most events and symptoms is clear  □ | Sequence of all events and symptoms is clear  □ |  |
| 1. *The PMH, FH, SH and ROS include pertinent elements related to active medical problems, allergies and meds* | Information has no clear connection to active medical problems  □ | Information adequately describes the patient’s active medical problems □ | Information completely and concisely describes all active problems □ |  |
| 1. *Presents focused physical exam, beginning with vitals, general statement and pertinent PE findings* | Information is disorganized or absent □ | Overall, physical exam is adequately presented □ | All relevant physical exam items are presented in an organized manner  □ |  |
| 1. *Lab studies/test results relevant to patient’s condition are clearly presented* | Irrelevant test results presented or significant results omitted □ | Minor omissions or a few extra results but overall adequately presented □ | All relevant results presented in an organized manner  □ |  |
| **SUMMARY STATEMENT and ASSESSMENT** |  |  |  |  |
| 1. *Begins with summary statement that synthesizes the critical elements of the patient’s history, physical exam and diagnostic studies into one sentence* | No summary statement or restatement of story w/out synthesis or relevant elements  □ | Most pertinent information synthesized; may repeat some unnecessary information  □ | Summary statement concisely synthesizes all key information  □ |  |
| 1. *Includes a differential diagnosis (DDx) for the patient’s condition(s) and problems* | No DDX given and/or focuses on trivial problems  □ | DDX given with several possibilities/most important problems prioritized  □ | Extensive DDX given, problems all appropriately prioritized  □ |  |
| **PLAN** |  |  |  |  |
| 1. *Identify patient at risk for becoming unstable* | Does not recognize basic signs of instability  □ | Recognizes patient as ill   □ | Recognizes patient as ill and identifies appropriate next step □ |  |
| 1. *Proposes a treatment plan that addresses issues raised by problems and/or diagnoses* | Plan is not relevant or related to the problem list  □ | Plan addresses most important issues/may omit active but lower priority problems  □ | Plan is complete and relates directly to problem list all active issues included  □ |  |
| ***Comments***  Meeting/exceeding expectations  □  □ | | | | |

Reviewed with student? ___Yes ___No
**Total** ____ /20
14/20 (70% passing)

Supplemental Figure 3: Oral Case Presentation Rating Scale for Outpatient OSCE

^1^Adapted from: King MA, Phillipi CA, Buchanan PM, Lewin LO. Developing Validity Evidence for the Written Pediatric History and Physical Exam Evaluation Rubric. *Acad Med*. 2017; 17 (1): 68-73.
