# Supplementary material for: Development and evaluation of a simulation-based transition to clerkship course
Source: Perspect Med Educ. 2020 May 26;9(6):379–84. doi: 10.1007/s40037-020-00590-4 (PMC7718359; doi:10.1007/s40037-020-00590-4)
Supplement: Supplementary file 4 — Supplemental Fig. 4: Outpatient OSCE Encounter Checklist by Standardized Patient [file 40037_2020_590_MOESM4_ESM.docx]

**Student: __________________________________________ Date:______________________**

**Standardized Patient: _______________________________**

**Transition to Clinical Experience OSCE Checklist**

**To be completed by Standardized Patient**

| **OSCE- Outpatient** | **No**  **(0)** | **Yes (1)** | **Comments** |
| --- | --- | --- | --- |
| **Did the student…** |  |  |  |
| 1. Knock before entering? |  |  |  |
| 1. Introduce her/himself by name? |  |  |  |
| 1. Identify his/her role or position? |  |  |  |
| 1. Wash or use hand sanitizer on hands? |  |  |  |
| 1. Ask about history that was relevant to the encounter? |  |  |  |
| 1. Avoid medical jargon? |  |  |  |
| 1. Make good eye contact? |  |  |  |
| 1. Did the student remain on task? |  |  |  |
| 1. Demonstrate an understanding of the reason for my visit and any concerns I (the patient) had? |  |  |  |
| 1. Summarize the visit in a way that I could understand? |  |  |  |
| 1. Use broad open-ended questions initially? |  |  |  |
| 1. Use a style that put me at ease? |  |  |  |
| 1. Listen attentively and display empathy? |  |  |  |
| 1. End the encounter and exit the room in a manner that was comfortable to me? |  |  |  |
| 1. Do you have significant concerns about the student’s ability to take a relevant history in a professional fashion? If yes, please comment. |  | | |

Total Pts (out of 14) ______
 10 needed for passing (70%)

Supplemental Figure 4: Outpatient OSCE Encounter Checklist by Standardized Patient
