# Supplementary material for: Development and evaluation of a simulation-based transition to clerkship course
Source: Perspect Med Educ. 2020 May 26;9(6):379–84. doi: 10.1007/s40037-020-00590-4 (PMC7718359; doi:10.1007/s40037-020-00590-4)
Supplement: Supplementary file 5 — Supplemental Table 1: Description of Transition to Clerkship Objective Structured Clinical Examination (OSCE) [file 40037_2020_590_MOESM5_ESM.docx]

| **Scenario** | **Description** | **Graded Tasks** |
| --- | --- | --- |
| Adult Inpatient | 66-year-old adult male/female admitted with an ST-elevation acute myocardial infarction (STEMI)­–seen in the emergency department (ED) and requiring progressive care from the ED to the cardiac catheterization lab and to the intensive care unit;. During scenario, student must participate in delivering care, recognize and propose solutions to patient deterioration and various other issues that arise. Student signs out what happened during their scenario to the next student | - Participation in a patient scenario that includes tasks that fall within the student physician’s limits of practice, including recognizing an unstable patient, calling for help appropriately and initiating basic life supportive care, maintaining safe environment for patients and colleagues, researching clinical questions, demonstrate proper use of personal protective equipment (PPE)/sterile gowning, and maintaining confidentiality (Supplemental Figure 1 - The student may also be asked to perform tasks outside of their scope of practice and have to speak up to their preceptor. |
| Pediatric Inpatient | Three-week-old female infant admitted with fever and rhinorrhea–requires lumbar puncture, which leads to meningitis diagnosis complicated by seizures and recurrent respiratory failure. During scenario, student must participate in delivering care, recognize and propose solutions to patient deterioration and various other issues that arise. Student signs out what happened during their scenario to the next student |  |
| Adult Outpatient | Pre-inpatient: 66-year-old adult male/female appears uncomfortable and in mild distress. Chief complaint is chest pain. Student takes a history and should ask to do a physical exam, before giving an oral case presentation to faculty  **OR**  Post-inpatient: 66-year-old adult male/female attends follow up after hospital discharge and notes pain/numbness in right hand where cardiac catheter had been previously inserted. Student takes a history and should ask to do a physical exam, before giving an oral case presentation to faculty | - A clear and concise oral case presentation to faculty, which should contain all of the required and pertinent information about the case, including that patient is at risk for becoming unstable and needs further, immediate treatment (Supplemental Figure 2) - An overall encounter evaluation from the standard patient (SP), which includes scoring and commentary about the student physician’s professionalism, efficiency, empathy and overall bedside manner (Supplemental Figure 3) |
| Pediatric Outpatient | Pre-inpatient: 32-year-old parent brings in three-week-old female infant with fever and runny nose. Student takes a history and should ask to do a physical exam, before giving an oral case presentation to faculty  **OR**  Post-inpatient: 32-year-old parent brings in five-week old female infant for follow up for hospitalization for meningitis and fears that child might be having seizures. Students takes a history and should ask to do a physical exam, before giving an oral case presentation to faculty |  |

**Supplemental Table 1: Description of Transition to Clerkship Objective Structured Clinical Examination (OSCE)**
