# Supplementary material for: Development and evaluation of a simulation-based transition to clerkship course
Source: Perspect Med Educ. 2020 May 26;9(6):379–84. doi: 10.1007/s40037-020-00590-4 (PMC7718359; doi:10.1007/s40037-020-00590-4)
Supplement: Supplementary file 6 — Supplemental Table 2: Means (and Standard Deviations) for Student Scores on the Self-Assessment Survey of Transition to Clerkship Objectives: Pre-versus Post-Course [file 40037_2020_590_MOESM6_ESM.docx]

**Supplemental Table 2. Means (and Standard Deviations) for Student Scores on the Self-Assessment Survey of Transition to Clerkship Objectives: Pre-versus Post-Course**

| **Survey Question^a^** | **Corresponding Course Goal** | **Pre-course**†† | **Post-course** †† | ***P* Value†** |
| --- | --- | --- | --- | --- |
| **Identifying an unstable adult patient** | 1 | 2.6 (0.87) | 4.0 (0.65) | <0.001 |
| **Identifying an unstable pediatric patient** | 1 | 2.0 (0.80) | 3.5 (0.76) | <0.001 |
| **Responding to an unstable patient** | 1 | 2.0 (1.00) | 4.1 (0.65) | <0.001 |
| **Basic Airway Management** | 1 | 2.2 (0.97) | 3.9 (0.74) | <0.001 |
| **Performing CPR** | 1 | 3.0 (1.00) | 4.2 (0.73) | <0.001 |
| **Taking a patient’s history** | 2 | 3.5 (0.81) | 4.2 (0.71) | <0.001 |
| **Giving an oral presentation** | 2 | 2.5 (0.84) | 3.5 (0.88) | <0.001 |
| **Giving a patient handoff** | 2 | 1.5 (0.75) | 3.0 (0.82) | <0.001 |
| **Assessing need for isolation in a hospitalized patient with an infectious disease** | 3 | 1.7 (0.77) | 3.2 (0.87) | <0.001 |
| **Choosing appropriate PPE^b^ for a patient with an infectious disease** | 3 | 2.1 (1.00) | 3.5 (0.88) | <0.001 |
| **Donning and doffing PPE^b^** | 3 | 2.6 (1.10) | 3.9 (0.81) | <0.001 |
| **Scrubbing, gowning and gloving for a sterile procedure** | 3 | 2.7 (1.30) | 3.8 (0.93) | <0.001 |
| **Handling and disposing of sharps** | 3 | 3.2 (1.20) | 4.4 (0.74) | <0.001 |
| **Performing a primary literature search** | 4 | 3.4 (0.94) | 4.1 (0.78) | <0.001 |
| **Assessing strengths and weaknesses of primary literature searches** | 4 | 2.9 (1.10) | 3.7 (0.94) | <0.001 |
| **Acknowledging personal limitations/strengths** | 4 | 3.7 (0.87) | 4.3 (0.73) | <0.001 |
| **Acquiring and utilizing feedback from superiors** | 4 | 3.7 (0.94) | 4.1 (0.78) | <0.001 |
| **Knowing when informed consent is needed** | 5 | 2.9 (0.90) | 3.8 (0.85) | <0.001 |
| **Obtaining informed consent** | 5 | 2.4 (1.10) | 2.9 (1.10) | <0.001 |
| **Maintaining patient confidentiality** | 5 | 4.3 (0.94) | 4.5 (0.69) | <0.001 |
| **Maintaining wellness in personal and professional life** | 6 | 3.4(1.10) | 3.5(1.10) | 0.345 |
| **Maintaining respectful and effective interprofessional team relationships** | 7 | 4.3(0.90) | 4.4(0.70) | 0.019 |
| **How useful was this course for preparing you for the clinical phase?** | - | - | 3.7(0.88)^c^ | - |

^a^ Question stem: “How comfortable are you with…” Applicable to all questions except final question

^b^ Personal Protective Equipment

^c^ Scale: 1=Not useful=1, 2=Barely, 3=Moderately, 4=Very, 5=Extremely; n=151

*Scale: 1=Not at all, 2=Slightly, 3=Moderately, 4=Mostly, 5=Completely; used for all questions except final question

†Wilcoxon signed-ranks (2-tailed)

††n=152 for all questions except final question
